# Enhanced Quantum Magnetometry with a Femtosecond Laser-Written Integrated Photonic Diamond Chip

**DOI:** 10.1021/acs.nanolett.5c00148

**Published:** 2025-04-21

**Authors:** Yanzhao Guo, Giulio Coccia, Vinaya Kumar Kavatamane, Argyro N. Giakoumaki, Anton N. Vetlugin, Roberta Ramponi, Cesare Soci, Paul E. Barclay, John P. Hadden, Anthony J. Bennett, Shane M. Eaton

**Affiliations:** † School of Engineering, 2112 Cardiff University, Queen’s Buildings, The Parade, Cardiff CF24 3AA, United Kingdom; ‡ Department of Physics, Politecnico di Milano, Piazza Leonardo da Vinci, 32, 20133 Milano, Italy; § Institute for Quantum Science and Technology, 2129 University of Calgary, Calgary, AB T2N 1N4, Canada; ∥ Centre for Disruptive Photonic Technologies, TPI, 54761 Nanyang Technological University, Singapore 639798, Singapore; ⊥ Translational Research Hub, 2112 Cardiff University, Maindy Road, Cardiff CF24 4HQ, United Kingdom; # 96976 Institute for Photonics and Nanotechnologies (CNR-IFN), Piazza Leonardo da Vinci, 32, 20133 Milano, Italy; ∇ Division of Physics and Applied Physics, SPMS, 54761 Nanyang Technological University, Singapore 639798, Singapore

**Keywords:** Quantum sensing, Femtosecond laser writing, Waveguide, Nitrogen-vacancy centers, Diamond

## Abstract

Ensemble negatively charged nitrogen-vacancy centers in diamond are promising quantum sensors. To optimize their sensitivity, it is crucial to increase the number of spins sampled and maximize their coupling to the detection system without degrading their spin properties. In this paper, we demonstrate enhanced quantum magnetometry via a buried laser-written waveguide in diamond with 4.5 ppm nitrogen-vacancy centers. The waveguide-coupled nitrogen-vacancy centers exhibit spin coherence properties comparable to those of nitrogen-vacancy centers in pristine diamond. Waveguide-enhanced magnetic field sensing is demonstrated in a fiber-coupled integrated photonic chip, where probing an increased volume of high-density spins results in 63 pT·Hz^–1/2^ of DC magnetic field sensitivity and 20 pT·Hz^–1/2^ of AC magnetic field sensitivity. This on-chip sensor realizes at least an order of magnitude improvement in sensitivity compared to the conventional confocal detection setup, paving the way for high-sensitivity quantum magnetometry with nitrogen-vacancy ensembles.

## Graphical abstract

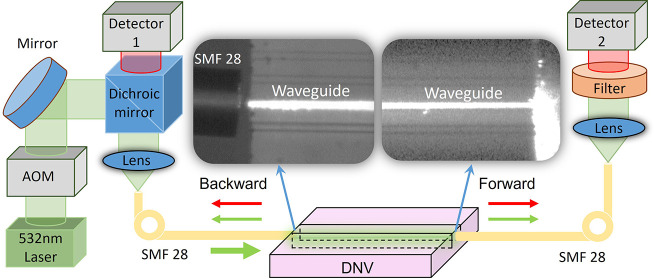

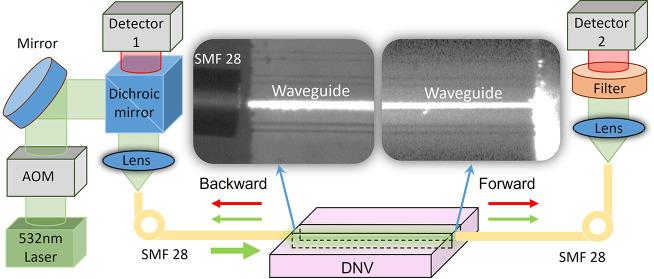

Quantum sensing with negatively charged nitrogen-vacancy centers (NVs) in diamond has attracted broad interest in the last two decades.^1,2^ Due to its asymmetric atomic structure,^3^ NVs are highly sensitive to weak external influences like temperature,^4^ pressure,^5,6^ electric field,^7^ and magnetic field^8^ at the nanoscale.^1^ Meanwhile, their long spin coherence time^9^ allows NV-based quantum sensors to achieve remarkable sensitivity.^2,10,11^ However, their relatively low optical excitation efficiency and finite photon collection efficiency have limited their sensitivity in practice.^2,12^ Although various submicrometer-integrated photonic structures for a single NV have been studied to enhance the photon collection rate,^13,14^ few can easily be applied to the ensemble of NVs, and most would degrade the coherence properties of NVs. In particular, given the sensitivity scales inversely with the square root of detected signal intensity,^2,8^ it is crucial to efficiently excite and collect from a large volume of ensemble NVs with good spin coherence properties.

Previous work on NV ensemble sensing was based on the millimeter size of diamond devices with subnanotesla sensitivities. For example, a light-trapping diamond waveguide geometry^15^ improves the probed number of NVs via increasing optical depth in a millimeter-sized bulk diamond or the integration of an optical fiber tip with a millimeter-sized diamond.^16^ Another solution could be to couple an ensemble of NVs into a fiber-integrated photonic structure, with confined fiber mode field-limited sensing resolution, for example, the waveguide integrated NVs in diamond.^17,18^ Recently, laser writing has been demonstrated as a powerful tool for fabricating photonic circuits with integrated quantum emitters from single to ensemble level.^19,20^ Moreover, laser-written waveguide-integrated NVs (WGINVs) have been shown to have comparable spin coherence properties to native NVs in diamond.^20^

In this paper, we demonstrate an on-chip fiber-integrated sensor with enhanced sensitivity via a laser-written waveguide in a diamond containing a 4.5 ppm density of NVs. The high-quality buried waveguides (type II geometry) are fabricated by femtosecond laser writing and exhibit insertion loss below 12 dB at 635 nm. We characterize the spectrum and the spin coherence properties of NVs in the waveguide and pristine regions, showing that waveguide fabrication does not degrade their photoluminescence (PL) emission or spin coherence time. A fiber–waveguide–fiber configuration is then demonstrated to show enhanced sensing of a DC magnetic field. Thanks to the probing of the ensemble NVs along the whole waveguide device, this setup achieves at least an order of magnitude improvement in sensitivity compared to the traditional confocal configuration. Moreover, by using an excitation and collection path in a buried waveguide,^19^ the probed target could be potentially placed on the diamond surface without direct laser excitation;^21^ therefore, our study provides a noninvasive way for future biosensing for samples vulnerable to optical illumination.^21,22^

Our study uses optical waveguides in a commercial chemical vapor deposition (CVD) diamond (DNV-B14) from Element 6 which has a uniform and high concentration of NVs (4.5 ppm of NVs with an inhomogeneous dephasing time *T*_2_^*^ of 0.5 μs at room temperature). As shown in Figure 1(a), optical waveguides consisting of pairs of parallel amorphous and graphitized modification lines were written by scanning laser pulses of a 515 nm wavelength, 300 fs pulse duration, and 500 kHz repetition rate across the sample using a 100× 1.25 NA oil immersion Olympus objective, 0.5 mm/s scan speed, 18 μm depth, and 13 μm separation between optical modification lines.^23^ There is no annealing after the laser writing fabrication. From left to right in Figure 1(b), we show four waveguides written with powers of 20, 30, 40, and 50 mW, where the overlaid mode profile in the cross-sectional image (lower plate) is the waveguide mode of a 635 nm test laser from a beam profiler. The mode parameters are detailed in Table 1. As the laser fabrication power was increased from 20 to 50 mW, we found that the insertion loss decreased and that the optical modes were more tightly confined. The insertion loss includes diamond intrinsic absorbance (see UV-Vis-NIR transmission spectrum of DNV-B14 diamond in Supporting Information (SI)), fiber–waveguide coupling loss, and waveguide propagation loss across the 2.8 mm chip. The waveguide written with laser power of 40 mW had an ∼5 μm mode field diameter (MFD) at 635 nm, and a 12 dB insertion loss is chosen for the further characterization of spectral properties, spin coherence properties, and magnetic field sensing.

**Figure 1:**
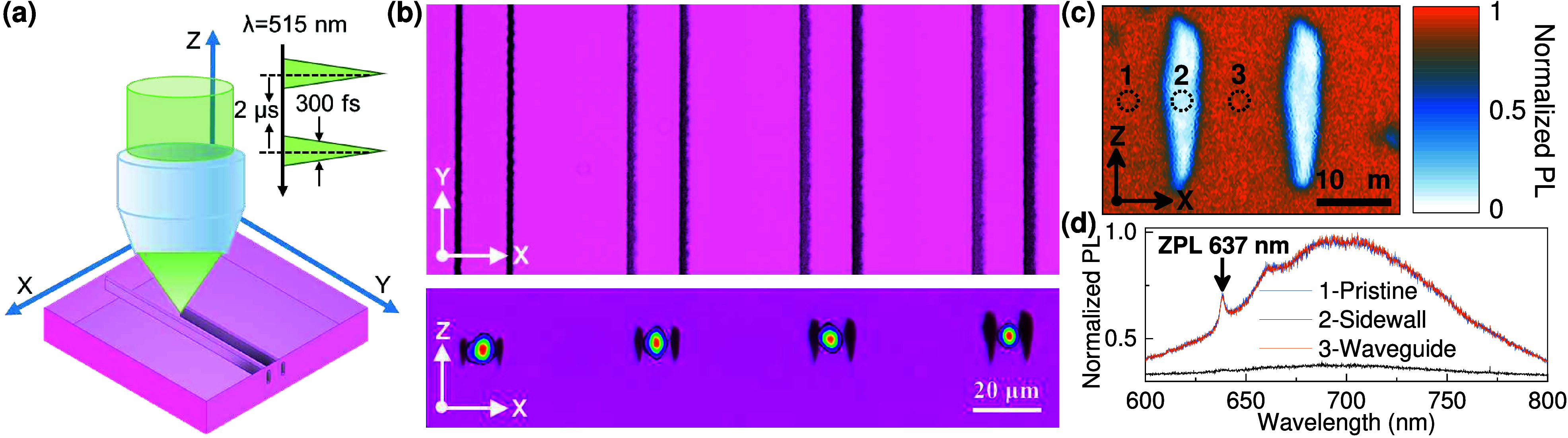
(a) Schematic diagram for the laser writing waveguides in a DNV-B14 diamond. (b) Overhead (upper plate) and cross-sectional (lower plate) optical microscopy images of waveguides with overlaid 635 nm mode profiles. (c) The confocal PL map of the WGINVs was written with a 40 mW femtosecond laser, mapped from the *y*-direction (along the waveguide). (d) PL emission spectrum for the 1-pristine, 2-sidewall, and 3-waveguide regions in a waveguide written with 40 mW.

**Table 1:** Insertion Loss and Mode Field Diameter at 635 nm for Laser-Written Waveguide in Diamond

Power (mW)	Insertion loss (dB)	MFD_*x*_ (μm)	MFD_*y*_ (μm)
20	14.2	5.5	6.0
30	12.1	4.3	6.2
40	11.6	4.8	5.5
50	11.6	4.2	4.9

We used a custom optically detected magnetic resonance (ODMR) confocal setup to characterize the waveguide’s spectrum and spin coherence properties. The setup is detailed in the SI. In Figure 1(c), measuring the sample from its facet edge, end on, we directly map the waveguide cross-section, which resolves three regions labeled as 1-pristine, 2-sidewall, and 3-waveguide regions. In Figure 1(d), the spectra of 1-pristine and 3-waveguide areas feature similar spectral properties with a clear 637 nm zero phonon line and broad phonon sideband extending to almost 800 nm. The PL intensity was reduced in the sidewall areas where the laser modification lines have converted the diamond to graphitized and amorphous carbon.^19^ Notably, the PL intensity and spectra in pristine and waveguide regions are identical, indicating the laser writing process has not degraded the NVs.

Spin coherence proprieties are core to quantum sensing, enabling the detection of weak perturbations to the spin’s environment. As a result of the NVs’ spin-selective transition, the spin coherence properties of its ***S*** = 1 triplet ground state can be easily characterized via ODMR techniques.^8^

In the absence of the magnetic field, strain, and electric field, the ground state’s *m*_*s*_ = ±1 sublevels are degenerate and separated by the axial zero-field splitting parameter *D* = 2870 MHz from the *m*_*s*_ = 0 sublevel which arises from the electron spin–spin interactions.^8^ These energy differences between the ground state sublevels can then be read out by recording NVs PL intensity while scanning the microwave frequency near resonance. This frequency domain zero-field ODMR contains information on microscopic local environment coupling with the NVs electron spin.^6^ In Figure 2(a), the zero-field continuous-wave (CW) ODMR in the pristine and waveguide regions exhibit an ∼3 MHz transverse zero-field splitting parameter of *E* due to the local strain and electric field from the diamond crystal.^8^ Additionally, the broader line width (*w*_1_ = 6.2 MHz and *w*_2_ = 7.8 MHz) of the *m*_*s*_ = ± 1 resonance peaks in the waveguide region indicates the increased nonhydrostatic strain induced by the laser-written fabrication process,^6,19^ compared to *w*_1_ = 5.7 MHz and *w*_2_ = 7.2 MHz observed in the pristine region.

**Figure 2:**
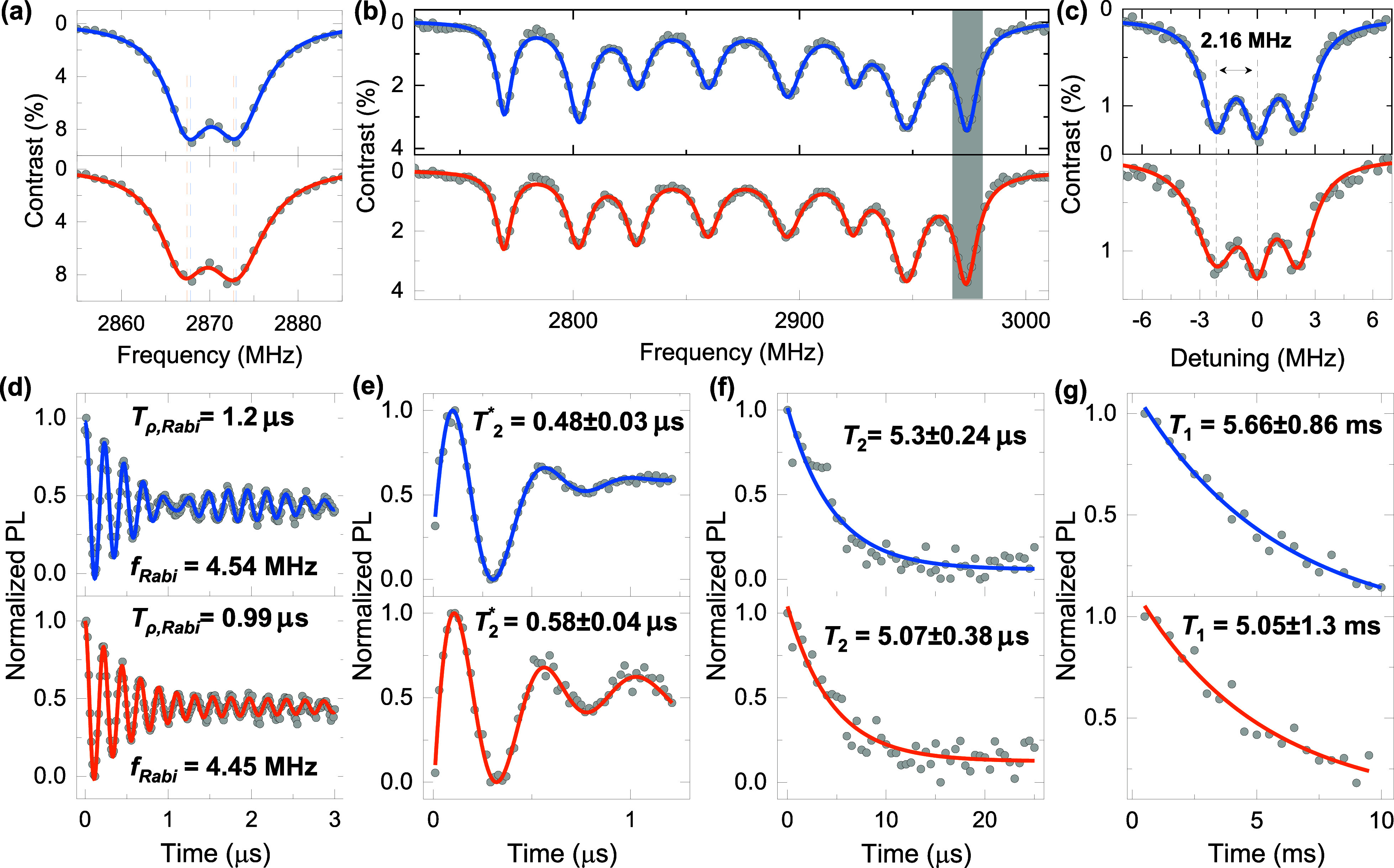
Spin coherence properties of an ensemble of NVs in the pristine (top) and waveguide (lower plates) regions. The gray points are the experimental data. The blue and orange lines are fitted curves. (a) Zero-field, CW ODMR. (b) CW ODMR with ∼5 mT applied magnetic field where the data are fitted by the multiple Lorenz equation. (c) Pulsed ODMR for the highest resonance transition in (b). (d–g) Rabi oscillation, free-induced decay, Hahn echo, and spin longitudinal relaxometry measurements, respectively, where the experimental data are fitted by exponential decay equations.^20,24^

An ∼5 mT magnetic field is applied to lift the degeneracy of the *m*_*s*_= ±1 transitions along the four NV orientations in Figure 2(b). The highest frequency transition is further investigated with pulsed ODMR as shown in Figure 2(c), where the typical 2.16 MHz ^14^N hyperfine splitting is resolved.

Electron spin Rabi oscillations, free-induction decay, Hahn-echo, and spin–lattice relaxometry of ensemble NVs in pristine and waveguide regions were measured with standard protocols^20^ as shown in Figure 2(d–g). The NV ensembles in both areas are shown to have comparable spin coherence times of Rabi oscillation decoherence time *T*_ρ,Rabi_ ∼ 1 μs, inhomogeneous dephasing time *T*_2_^*^∼ 0.5 μs, spin transverse relaxation time *T*_2_ ∼ 5 μs, and longitudinal relaxation times *T*_1_ ∼ 5 ms. This implies that laser writing fabrication does not degrade the spin coherence properties of the ensemble.^20^ This should be contrasted with other fabrication methods that have been used to create photonic structures in diamond, such as plasma etching^14,25^ and focused-ion beam,^26^ which have been shown to have a detrimental effect on these parameters.^2,14^

Compared to the conventional free space confocal setup for limited numbers of NVs, a fiber–waveguide–fiber configuration was used for high-density ensemble waveguide integrated NVs in Figure 3(a). Two SMF-28 single-mode fibers were used to couple to opposing facets to probe NVs along the entire waveguide efficiently. The input fiber delivers green laser excitation and collects the backward traveling red fluorescence from the NVs in the waveguide, which is filtered with a dichroic splitter and directed to the first detector. The second fiber solely collects the forward-traveling red fluorescence from the waveguide and directs it to a second detector. Detailed information on the fiber–waveguide–fiber setup is available in the SI.

**Figure 3:**
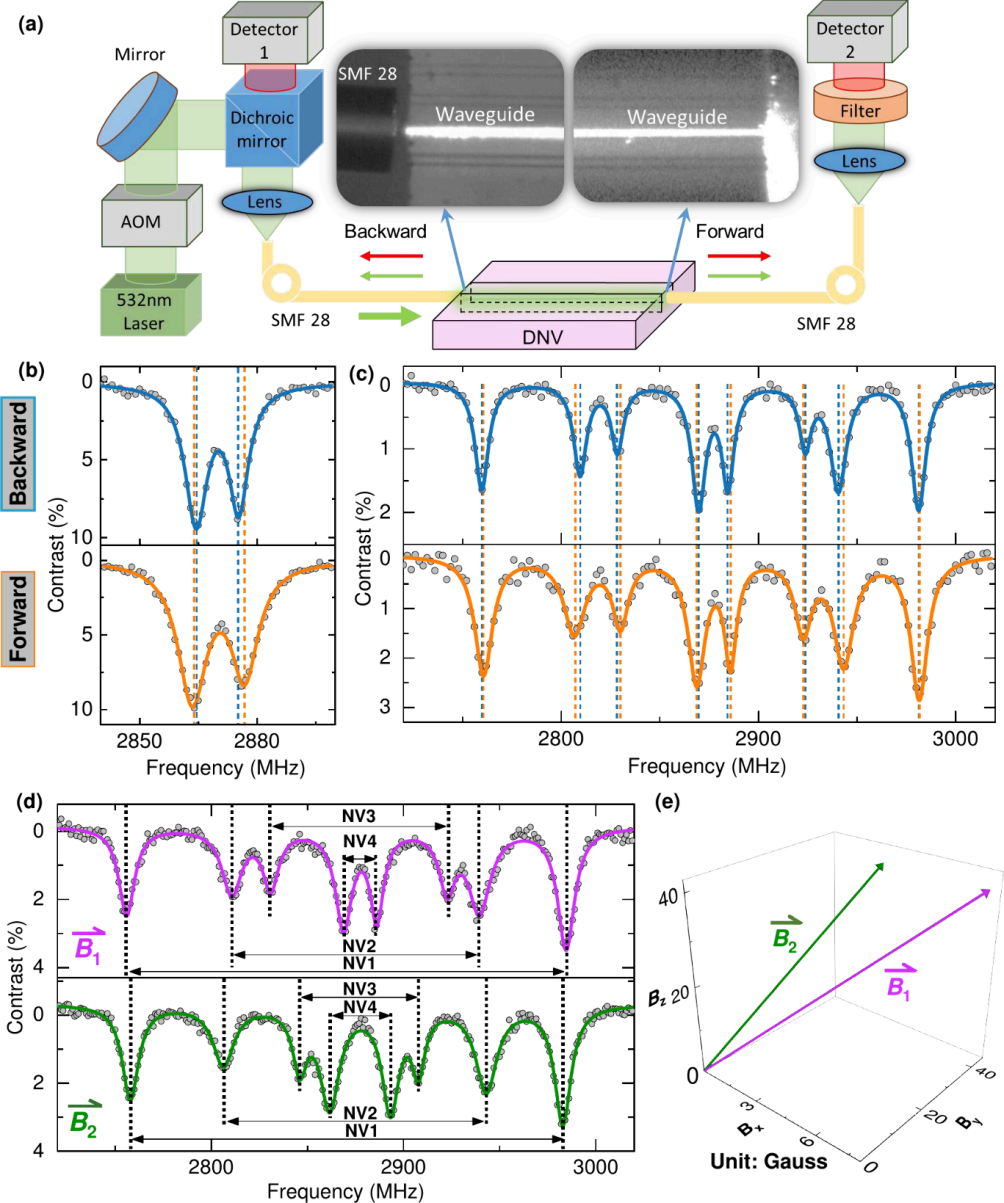
Enhanced quantum sensing via fiber–waveguide–fiber configuration. (a) Schematic diagram of fiber–waveguide–fiber configuration where the inset two microscopy images show backward and forward travel light in the waveguide. (b) Zero-field ODMR recorded by backward (upper plates) and forward (lower plates) PL. (c) ODMR with applied magnetic field is recorded by backward (upper plates) and forward PL (lower plates), where the vertical blue and orange dashed lines represent the resonance frequencies from forward and backward traveling ODMR curve fitting. (d) Magnetic field sensing via the forward traveling waveguide ODMR, where vertical black lines are the resonance frequencies responding to probed magnet fields $\overset{⇀}{B_{1}}$ and $\overset{⇀}{B_{2}}$ . (e) Magnetic field vector in Cartesian coordinates inferred from (d).

Figure 3(b) shows the zero-field ODMR recorded by using the backward and forward traveling PL emission from the chip. Compared to the confocal zero-field ODMR in Figure 2(a), the larger transverse zero-field splitting parameters *E* (6.7 MHz for backward ODMR and 5.4 MHz for forward ODMR) are observed in the fiber–waveguide–fiber configuration in Figure 3(b). These larger splitting parameters *E* implies the average effect of the strain-induced and electric field-induced splitting for the ensemble NVs along the whole waveguide region.^17^ Moreover, the 10% ODMR contrast suggests an excellent ODMR response, potentially leading to high sensitivity. Additional laser power and microwave power-dependent ODMR are included in the SI. We also observed an ODMR response with applied magnetic field in Figure 3(c) using the forward and backward traveling fluorescence from the chip. Eight resonance peaks resulting from the four different NVs orientations were clearly resolved. The contrast of the highest frequency peak in the ODMR spectrum is over 3%, comparable to the confocal ODMR results. Meanwhile, there are also some resonance frequency shifts between forward and backward ODMR. This potentially could be used to determine the magnetic field gradient along the waveguide length of 2.8 mm, but is beyond the scope of this work.

By solely taking forward traveling PL, as shown in Figure 3(d), applied magnetic fields $\overset{⇀}{B_{1}}$ and $\overset{⇀}{B_{2}}$ are probed by tracking four distinct Zeeman split pairs of peaks originating from four different ⟨111⟩ NV_*i*_ orientations. The magnetic field projection *B*_*i*_ along the NV*_i_* is calculated by the Zeeman effect in the ground state via the equation,^8^

$$\nu_{\pm }\left(B_{i}\right)=D\pm \sqrt{{\left(\frac{g\mu_{B}}{h}B_{i}\right)}^{2}+E^{2}}$$ 1

where ν_±_(*B*_*i*_) is the ODMR resonance frequency for different Zeeman splittings from four different NV orientations, *g* ∼ 2.0 is the Landé *g*-factor, μ_*B*_ is the Bohr magneton, and *h* is the Planck constant. As shown in Figure 3(e), we inferred the magnetic field vectors $\overset{⇀}{B_{1}}$ and $\overset{⇀}{B_{2}}$ as shown in Figure 3(d) via simple geometric arguments to transform the tetrahedral directions into Cartesian coordinates.^27^ We note the magnetic field vector in lab coordinates can be defined by the excitation polarization plot for four differently oriented NVs.^28,29^

The intrinsic sensitivity is not only dependent on the strong response to the target signal but also on avoiding interactions with undesirable noise.^30^ Generally, the photon-shot-noise-limited DC (η_dc_) and AC (η_ac_) sensitivities can be used to quantify the sensitivity and are given by^8^

$$\eta_{\mathrm{dc}}∼\frac{\hbar }{g\mu_{B}}\frac{1}{\Lambda \sqrt{Ct_{L}}}\times \frac{1}{\sqrt{T_{2}^{*}}}$$ 2

$$\eta_{\mathrm{ac}}=\eta_{\mathrm{dc}}\sqrt{\frac{T_{2}^{*}}{T_{2}}}$$ 3

respectively, where *ℏ* is the reduced Planck constant, and *t*_*L*_ ∼ 0.5 μs is the readout duration time. *T*_2_^*^ is ∼0.5 μs, and *T*_2_ is ∼5 μs where the spin coherence properties of the WGINVs are consistent with that of native NVs in pristine regions. Λ ∼ 3% is ODMR contrast. *C* is the total detected PL rate, relying on the excitation power and experiment configuration. For a conventional top-down confocal configuration with a NA = 0.9 objective, the saturation PL rate of 362 GHz and saturation power of 11.7 mW are achieved, resulting in η_dc_ = 627 pT·Hz^–1/2^ and η_ac_ = 198 pT·Hz^–1/2^.^8^ The power-dependent PL data are in the SI.

In the fiber–waveguide–fiber configuration, the saturation PL rate is affected by a number of factors such as the (i) enlarged mode area with a diameter of 5 μm in the waveguide as shown in Figure 1(b) and Table 1 relative to the confocal spot with a diameter of 0.5 μm as shown in Appendix C in our previous work.^20^ This increases the number of NVs probed in the unit optical plane and the saturation laser power, by a factor of ∼100. The detailed mode profile comparison is shown in Figure S1 in the Supporting Information. (ii) There is an increased optical depth of NVs probed by the beam as it propagates along the waveguide, relative to the Rayleigh range of the excitation volume in the confocal configuration.^20^ (iii) The waveguides have a reduced numerical aperture, arising from their weak confinement, of the order of NA = 0.012 ^18^ compared to a NA in the confocal system of NA ∼ 0.4 inside the diamond. (iv) There is a coupling loss due to mode mismatch between the fiber and waveguide.

We experimentally assess how these effects combine to scale the PL rate in the fiber–waveguide–fiber configuration. A 10-fold larger mode field diameter in the waveguide requires 100 times higher excitation power to reach saturation,^18,20^ which at 1.17 W is outside the range of our experimental apparatus. In this case, a series of laser powers up to 1.5 mW, much less than saturation power, is used to evaluate the PL emission comparison in the fiber–waveguide–fiber configuration and confocal configuration (Figure S1 in Supporting Information). These two configurations result in comparable photon detection rates within this laser power range. For example, under 1.5 mW laser power, we observed 3.80 GHz PL rate in the forward direction and a 40.1 GHz PL rate in the backward-traveling fluorescence which is comparable to the PL emission of 42.7 GHz in the confocal configuration under the same laser power. This indicates that the fiber–waveguide–fiber configuration achieves comparable PL emission to the confocal setup by probing a larger NV volume, despite its 100 times enlarged excitation area reducing excitation laser power density by 100.^20^ Therefore, we can estimate that fiber–waveguide–fiber configuration increases the saturation power and saturation PL rate by a factor of 100, offering a 10-fold improvement of sensitivity of η_dc_= 63 pT·Hz^–1/2^ and η_ac_ = 20 pT·Hz^–1/2^ compared to the confocal configuration.

This level of sensitivity makes this waveguide geometry quantum sensor competitive with other platforms in the ∼10 μm length scale such as superconducting quantum interference devices and Hall sensors.^31^ Furthermore, the possibility of integrating microfluidic channels^19^ for ion transport and neuron imaging^21^ is attractive for this technology. Moreover, the sensitivity could also be improved an order of magnitude by an optimal sensing protocol^11^ and a hybrid-enhanced sensing device.^10^

We have introduced an approach for waveguide-enhanced quantum sensing using a high-quality buried waveguide in a diamond chip with 4.5 ppm of NV density. We show that femtosecond laser writing does not change the density, spectrum, and spin coherence properties of the NVs, leading to a high sensitivity and an increased optical depth. In a fiber–waveguide–fiber setup, we obtained a robust ODMR response from which we inferred the magnetic field magnitude and direction. The increased NV density in this chip, combined with the advantages of using a fiber–waveguide coupled system leads to η_dc_ = 63 pT·Hz^–1/2^ and η_ac_ = 20 pT·Hz^–1/2^. Other than the improved sensitivity, WGINVs in DNV-B14 are significantly more reproducible compared to the WGINVs in high-pressure high-temperature diamond in our previous report,^20^ thanks to a more uniform NV density in the CVD produced sample. Future work will focus on applying this technology to electric field sensing or biosensing. Alternative photonic structures with integrated NVs could be tailored for different applications by leveraging the adaptability of the femtosecond laser direct-write process.

## Supplementary Material



## Data Availability

Data supporting the findings of this study are available in the Cardiff University Research Data Repository at https://doi.org/10.17035/cardiff.28796201.

## Notes

Experimental setup details, UV-Vis-NIR absorptance for diamond and laser and microwave power-dependent ODMR spectra for NVs (PDF)
