# Supplementary material for: Enhanced Quantum Magnetometry with a Femtosecond Laser-Written Integrated Photonic Diamond Chip
Source: Nano Lett. 2025 Apr 21;25(20):8096–102. doi: 10.1021/acs.nanolett.5c00148 (PMC12100711; doi:10.1021/acs.nanolett.5c00148)
Supplement: Supplementary file 1 [file nl5c00148_si_001.pdf]

Supporting Information for Enhanced Quantum Magnetometry with a Femtosecond Laser-written Integrated Photonic Diamond Chip

Yanzhao Guo,[*,†,‖] Giulio Coccia,[‡,⊥] Vinaya Kumar Kavatamane,[¶] Argyro N. Giakoumaki,[‡,⊥] Anton N. Vetlugin,[§,#] Roberta Ramponi,[‡,⊥] Cesare Soci,[§,#] Paul E. Barclay,[¶] John P. Hadden,[†,‖] Anthony J. Bennett,[*,†,‖] and Shane M. Eaton[‡,⊥]

†*School of Engineering, Cardiff University, Queen's Buildings, The Parade, Cardiff, CF24 3AA, United Kingdom*

‡*Department of Physics, Politecnico di Milano, Piazza Leonardo da Vinci, 32, 20133 Milano, Italy*

¶*Institute for Quantum Science and Technology, University of Calgary, Calgary, AB T2N 1N4, Canada*

§*Centre for Disruptive Photonic Technologies, TPI, Nanyang Technological University, Singapore*

‖*Translational Research Hub, Cardiff University, Maindy Road, Cardiff, CF24 4HQ, United Kingdom*

⊥*Institute for Photonics and Nanotechnologies (CNR-IFN), Piazza Leonardo da Vinci, 32, 20133 Milano, Italy*

#*Division of Physics and Applied Physics, SPMS, Nanyang Technological University, Singapore*

E-mail: GuoY65@cardiff.ac.uk; BennettA19@cardiff.ac.uk

Confocal setup

A continuous-wave (CW) 532 nm crystal laser was modulated by an acoustic-optic modulator (ISOMET 553F-2) with < 10 ns rise and fall time. A 2-axis Galvo mirror (GVS002) and 100 $\times$ Nikon objective with NA=0.9 were integrated into a 4f imaging system for 2D x-y scanning. Depth scanning (z) was implemented by a motorized sample stage. The PL was optically filtered by the dichroic mirror, 532 nm long-pass filter, and 650 nm long-pass filter, before detection on SPCM-AQRH silicon avalanche photodiodes (Excelitas) or a spectrometer with a silicon CCD. The optional ND filter is also used to keep the PL rate within the APD's linear response range (2 MHz) for the power-dependent PL saturation measurement in Fig. S1(a). The microwave (MW) field is generated by an E4438B MW source, modulated by a RF switch (ZASWA-2-50DRA+) and amplified by a MW amplifier (ZHL-42W+), and eventually transmitted to the sample by a patch antenna. The optically detected magnetic resonance (ODMR) spectroscopy, Rabi oscillation, free induction decay, Hahn echo, and T_1 measurements are implemented by the standard protocol.

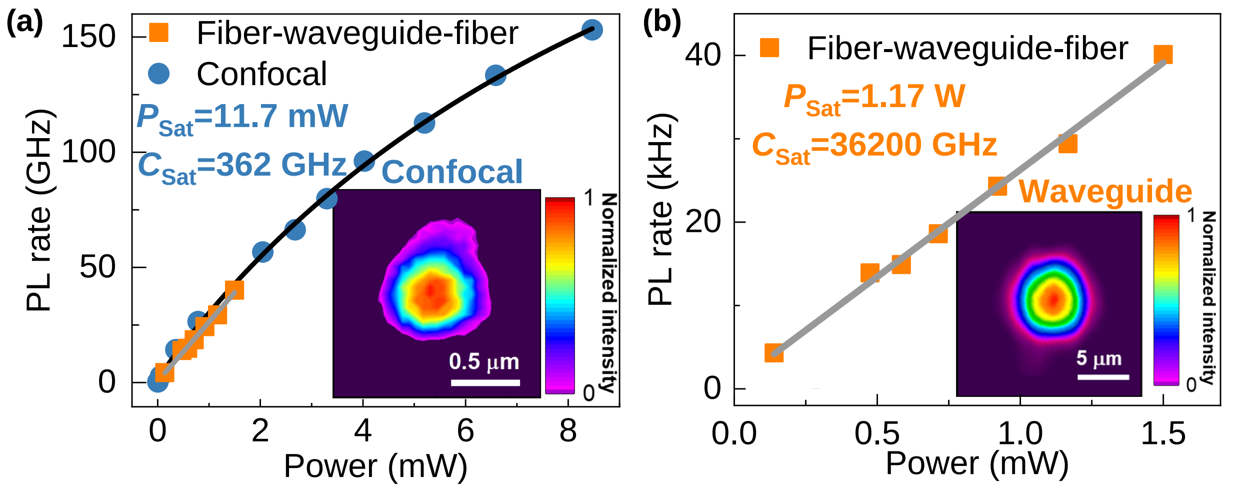

Figure S1: (a) Power-dependent PL rate for confocal and backward emission in fiber-waveguide-fiber configuration, where the insert image is the confocal mode field. (b) The power-dependent PL rate in fiber-waveguide-fiber configuration replotted from (a), where the insert image is the waveguide mode field.

UV-Vis-NIR transmission spectrum for pristine DNV-B14 diamond

The UV-Vis-NIR spectra of the DNV-B14 diamond and IIa diamond were measured using an MSV-5200 Microspectrophotometer. In Fig. S2, compared to high pure IIa diamond, we observed non-negligible loss in DNV-B14 diamond.

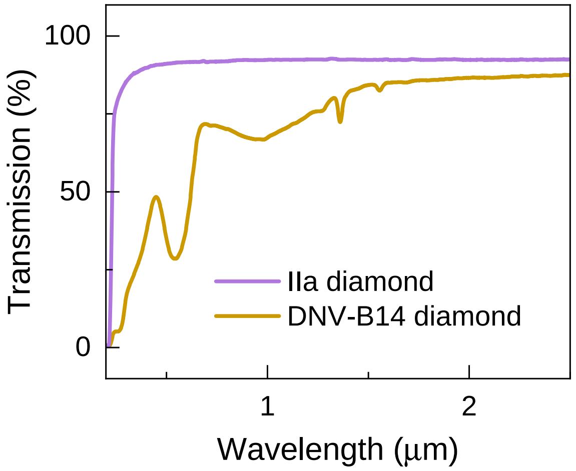

Figure S2: UV-Vis-NIR transmission spectra for pristine DNV-B14 diamond and IIa diamond

Fiber-waveguide-fiber configuration

In the fiber-waveguide-fiber configuration, the input laser is coupled into a fiber polarization controller for polarization tuning, then connected to SMF-28 Ultra single-mode fiber, coupled into waveguide in diamond. The green laser would excite the NVs along the waveguide which transmit the PL emission and the green laser along the waveguide. Another SMF-28 Ultra single-mode fiber, as output fiber, would extract the waveguide mode of PL emission and green laser into the fiber space again where the PL emission and green laser are separated by the 532 nm and 650 nm long pass filter in free space. The APD and optional ND filters are used to read out the signals. The power-dependent PL saturation measurement in Fig. S1(b).

Power dependent zero field ODMR

In confocal microscopy, the laser power and MW power greatly impact the ODMR shape. This is typical due to the non-negligible power broadening and dynamics completion between laser polarization and MW manipulation in the ground state. In the fiber-waveguide-fiber configuration, from Fig. S3, the laser excitation power appears to play less role than the microwave in the ODMR spectrum recorded by forward and backward traveling PL. This behavior might be because the excited laser powers are far less than the saturation power for fiber-waveguide-fiber configuration.

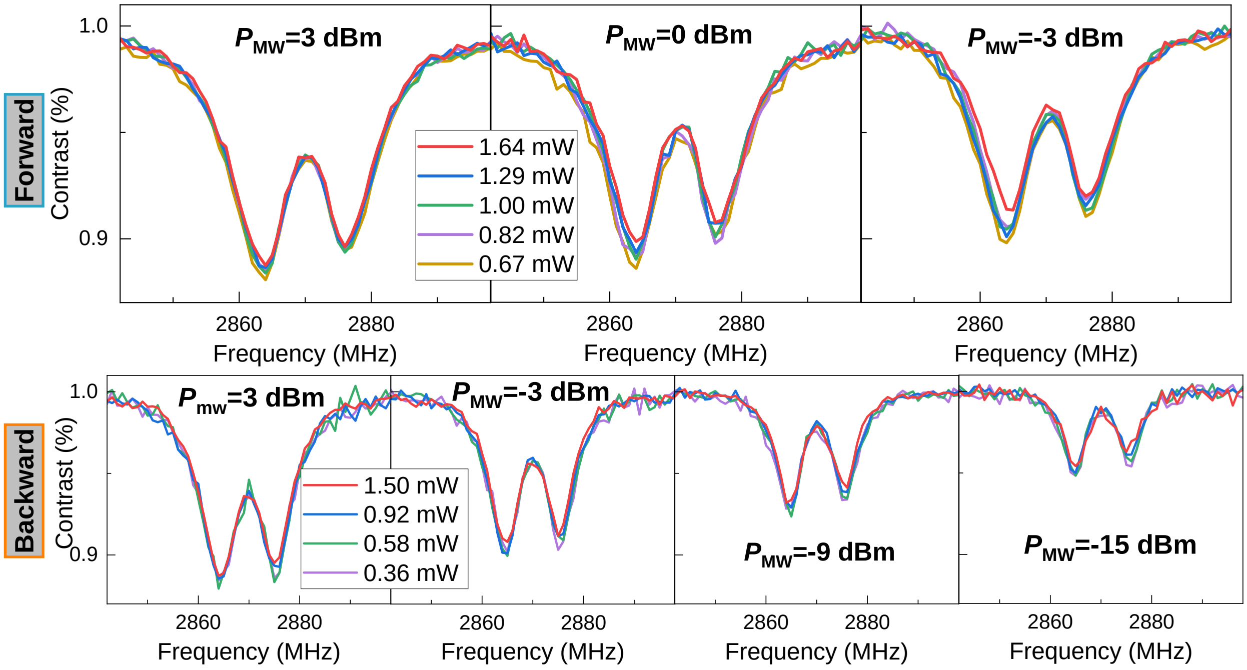

Figure S3: The laser power and microwave power-dependent ODMR, where P_{MW} is the MW power from the direct output of E4438B MW source
